# Impaired T-cell response to phytohemagglutinin (PHA) in tuberculosis patients is associated with high IL-6 plasma levels and normalizes early during anti-mycobacterial treatment

**DOI:** 10.1007/s15010-023-01977-1

**Published:** 2023-01-18

**Authors:** Monika M. Vivekanandan, Ernest Adankwah, Wilfred Aniagyei, Isaac Acheampong, Difery Minadzi, Augustine Yeboah, Joseph F. Arthur, Millicent Lamptey, Mohammed K. Abass, Francis Kumbel, Francis Osei-Yeboah, Amidu Gawusu, Linda Batsa Debrah, Dorcas O. Owusu, Alexander Debrah, Ertan Mayatepek, Julia Seyfarth, Richard O. Phillips, Marc Jacobsen

**Affiliations:** 1 Kumasi Centre for Collaborative Research in Tropical Medicine (KCCR), Kumasi, Ghana; 2 Department of Medical Diagnostics, College of Health Sciences, Kwame Nkrumah University of Science and Technology (KNUST), Kumasi, Ghana; 3 Agogo Presbyterian Hospital, Agogo, Ghana; 4 St. Mathias Catholic Hospital, Yeji, Ghana; 5 Atebubu District Hospital, Atebubu, Ghana; 6 Sene West Health Directorate, Kwame Danso, Ghana; 7 Department of General Pediatrics, Neonatology and Pediatric Cardiology, Medical Faculty, University Hospital Duesseldorf, University Children’s Hospital, Heinrich-Heine University, Moorenstr. 5, 40225 Duesseldorf, Germany; 8 School of Medicine and Dentistry, College of Health Sciences, KNUST, Kumasi, Ghana

**Keywords:** Phytohemaglutinin, Tuberculosis, IFN-γ, T-cell cytokines, Interleukin-6, Plasma milieu

## Abstract

**Purpose:**

Human tuberculosis is characterized by immunopathology that affects T-cell phenotype and functions. Previous studies found impaired T-cell response to phytohemagglutinin (PHA) in patients with acute tuberculosis. However, the influence of disease severity, affected T-cell subsets, and underlying mechanisms remain elusive.

**Methods:**

Here we investigated PHA-induced and antigen-specific T-cell effector cytokines in tuberculosis patients (*n* = 55) as well as in healthy asymptomatic contacts (*n* = 32) from Ghana. Effects of *Mycobacterium (M.) tuberculosis* sputum burden and treatment response were analyzed and compared during follow-up. Finally, cytokine characteristics of the aberrant plasma milieu in tuberculosis were analyzed as a potential cause for impaired PHA response.

**Results:**

PHA-induced IFN-γ expression was significantly lower in sputum-positive tuberculosis patients as compared to both, contacts and paucibacillary cases, and efficiently discriminated the study groups. T-cell responses to PHA increased significantly early during treatment and this was more pronounced in tuberculosis patients with rapid treatment response. Analysis of alternative cytokines revealed distinct patterns and IL-22, as well as IL-10, showed comparable expression to IFN-γ in response to PHA. Finally, we found that high IL-6 plasma levels were strongly associated with impaired IFN-γ and IL-22 response to PHA.

**Conclusion:**

We conclude that impaired T-cell response to PHA stimulation in acute tuberculosis patients (i) was potentially caused by the aberrant plasma milieu, (ii) affected differentially polarized T-cell subsets, (iii) normalized early during treatment. This study shed light on the mechanisms of impaired T-cell functions in tuberculosis and yielded promising biomarker candidates for diagnosis and monitoring of treatment response.

**Supplementary Information:**

The online version contains supplementary material available at 10.1007/s15010-023-01977-1.

## Introduction

Tuberculosis remains a major health threat with approximately 10 million new cases and 2 million deaths occurring annually [1]. *Mycobacterium tuberculosis* infection progresses towards acute disease in approximately 10% of index patient contacts, however, it remains asymptomatic in the vast majority of individuals due to effective immune surveillance [2]. T cells are of central importance for host immunity and T helper type 1 (T_H_1) cells coordinate innate and adaptive immune effector functions leading to protection against tuberculosis disease progression [2]. Detection of IFN-γ produced by T_H_1 cells, after in vitro stimulation with *M. tuberculosis* specific antigens, forms the basis for immune-based tests (i.e., IFN-γ release assays, IGRAs), which contribute to diagnosis of tuberculosis. IGRA sensitivity was found to be low in tuberculosis patients from Ghana as a consequence of low IFN-γ expression [3, 4]. Besides impaired antigen-specific T-cells, decreased T-cell responses to PHA, which serves as a positive control in IGRA tests has been observed [5–7]. Low PHA-induced T-cell responses often lead to indeterminate IGRA results in tuberculosis patients [3, 5]. Previous studies from our group indicated that analysis of alternative cytokines can improve measurement of antigen-specific T-cell response [3, 4]. However, if measurement of alternative cytokines can be used to circumvent impaired PHA-induced IFN-γ response in tuberculosis patients is not yet known. Besides its effect on IGRA results, low PHA response of tuberculosis patients has been shown to qualify as a biomarker for discrimination between latent *M. tuberculosis* infection and acute tuberculosis [6, 7]. Especially a combination of antigen-specific and PHA-induced IFN-γ expression from IGRAs (i.e., antigen/PHA ratios) gained promising results [6–10]. In addition, a recent study indicated potential capacity of antigen/PHA ratios to monitor efficacy of treatment [11].

Here, we compared antigen-specific and PHA-induced IFN-γ expression in tuberculosis patients with differential *M. tuberculosis* sputum burden and contacts. Association with treatment response was investigated and alternative cytokines produced by T cells in response to PHA were determined. Finally, potential causative plasma cytokines were determined for association with impaired PHA T-cell responses.

## Materials and methods

### Study cohorts and clinical characterization

We recruited tuberculosis patients (*n* = 55) and asymptomatic potentially *M. tuberculosis* infected contacts (contacts, *n* = 32) from April 2019 to September 2021 at the Agogo Presbyterian Hospital, the St. Mathias Catholic Hospital, the Atebubu District Hospital, and the Sene West District Hospital in Ghana. Diagnosis of active tuberculosis was based on patient history, clinical examination, chest X-ray, as well as sputum tests for acid-fast bacilli. All patients had chest X-ray and clinical symptoms suggestive of tuberculosis. Laboratory tests (i.e., sputum smear, culture, GeneXpert) confirmed diagnosis for all tuberculosis patients (Supplementary Fig. 1, Table 1). Tuberculosis patients with negative sputum smear and culture results at baseline (BL) were termed ‘paucibacillary’ (TB-PB; *n* = 11). These TB-PB patients were either microbiological confirmed by GeneXpert analysis at baseline (*n* = 7) or by sputum smear/culture analyses during treatment (*n* = 4). Presumptive tuberculosis patients without microbiological confirmation were excluded. Individual laboratory parameters of all tuberculosis patients are provided as Supplementary Table 1. Differential diagnosis of non-tuberculous mycobacterial (NTM) infection is not performed routinely in the participating hospitals. Hence, we cannot exclude NTM infections in participating tuberculosis patients. It was the first known episode of tuberculosis for all included patients. Patient characteristics are summarized in Table 1. All patients were included prior to initiation of treatment and blood was taken at BL, 6 weeks as well as 16 weeks thereafter for plasma analyses. The study design including criteria for classification of tuberculosis patients as ‘rapid’ (TB-RR) or ‘slow’ (TB-SR) treatment responder (only for TB-SP) and paucibacillary cases is depicted in Supplementary Fig. 1. Since not for all patients with tuberculosis sputum results at 6 weeks were available (*n* = 5), sputum analyses after 9 and 12 weeks were included for classification (Supplementary Fig. 1). TB-RR had no positive sputum test after 9 weeks, TB-SR had at least one positive test after week 9. Laboratory tests were done for all time points. Contacts showed no symptoms of tuberculosis but were close relatives living in the same household with indexed tuberculosis patients according to self-report and direct observation. We used selection criteria as for previous studies in the same Ghanaian population, which confirmed *M. tuberculosis* infection for comparable cohorts [4, 5]. The characteristics of the study participants are presented in Table 1.

**Table 1:** Characteristics of study participants

	TB-SP*,**	TB-PB	Controls
Number, *n*	44	11	32
Age, (mean, SD)	50.4 ± 14.8	38.3 ± 13.5	46.9 ± 15.1
Males *n* (%)	27 (61.3)	7 (63.6)	20 (62.5)
GeneXpert positive *n* (%)	39^δ^ (88.6)	7 (63.6)	na
Culture positive *n* (%)	39^#^ (88.6)	4 (36.4)***	na
Chest X-ray (suggestive of TB) *n* (%)	44 (100)	11 (100)	na
Symptoms			
Cough > 2 wks, *n* (%)	42 (95.5)	6 (54.5)	na
Fever, *n* (%)	12 (27.3)	2 (18.2)	na
Chest pain, *n* (%)	33 (75.0)	5 (45.5)	na
Hemoptysis, *n* (%)	9 (20.5)	1 (9.1)	na
Weight loss, *n* (%)	22 (50.0)	4 (36.4)	na

^*^Plasma and CBA cytokine analyses were done on a subgroup of age- and sex-matched TB-SP patient samples (*n* = 30). **Out of the 44 TB-SP, 40 had complete data for all timepoint (22 TB-RR and 18 TB-SR) and were used for TB-SP subgroup and time course analyses. ***Four TB-PB with missing/negative GeneXpert results (see Supplementary Table 1) were confirmed by sputum smear/culture during treatment. ^#^5 out of 44 patients were culture negative but GeneXpert positive and had chest X-ray findings suggestive of tuberculosis. ^δ^5 patients had missing data for GeneXpert. na, not applicable

### Whole blood stimulation assays and quantification of IFN-γ

Whole blood (100 µl) was cultured in 100 μl RPMI supplemented with Penicillin/streptomycin (100 U/ml) and L-glutamine (2 mM) using a 96-well U bottom plate. Samples were stimulated with recombinant ESAT6-CFP10 fusion protein (2 µg/ml) (kindly provided by Tom Ottenhoff, Leiden University Medical Center), purified protein derivative of *Mtb* (PPD_Mtb_; 10 µg/ml; Statens Serum Institute, Denmark), phytohemagglutinin (PHA; 10 µg/ml; Sigma-Aldrich, USA) or left unstimulated for 20 h at 37 °C and 5% CO_2._ The supernatants were harvested and stored at -80 °C until further analysis.

**Fig. 1:**
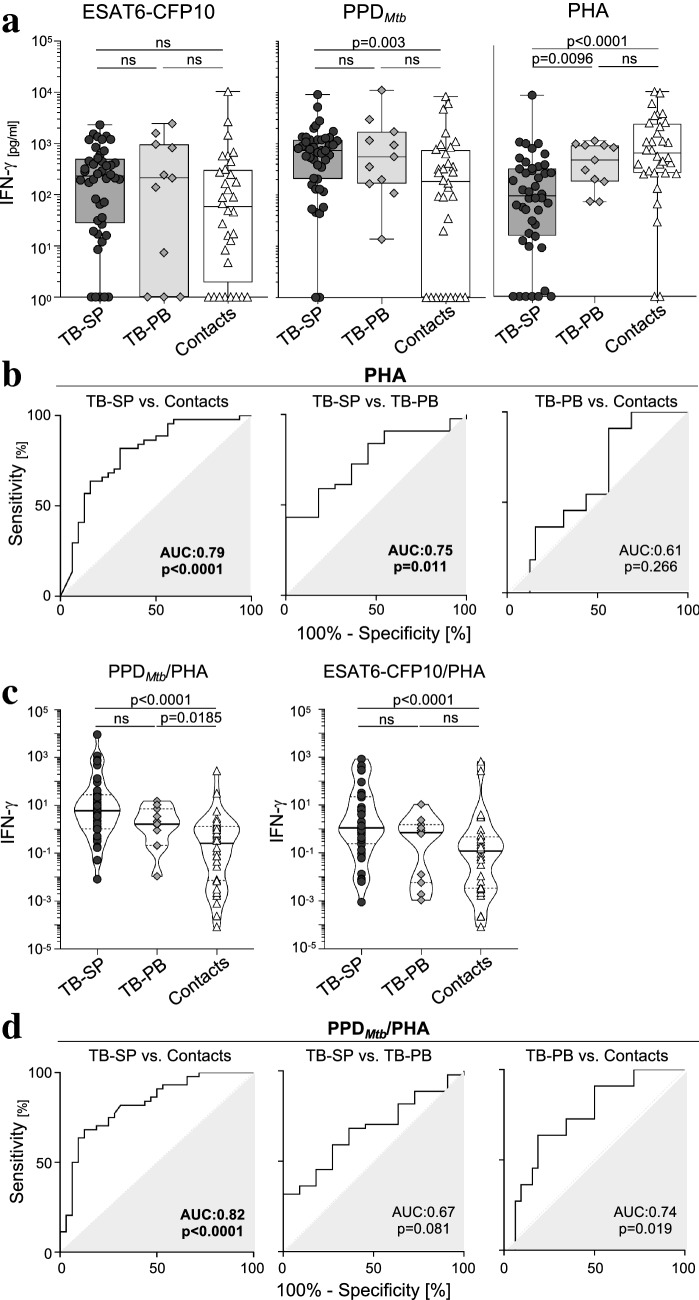
Impaired PHA response in tuberculosis patients with high *M. tuberculosis* burden. IFN-γ concentrations in supernatants of samples after overnight antigen-specific (i.e., PPD_Mtb_, ESAT6-CFP10) or PHA (i.e., phytohemagglutinin) stimulation from tuberculosis patients with high (TB-SP; dark grey circles and boxes; *n* = 44) and low (TB-PB; bright grey diamonds and boxes; *n* = 11) *M. tuberculosis* burden as well as contacts (open triangles and boxes; *n* = 32) are shown. (**a**) Combined symbol/box and whisker graph depicts individual concentrations of IFN-γ as well as study group medians and ranges. (**c**) Violin plots depict individual ratios of antigen-specific/PHA-induced IFN-γ expression as well as study group distributions including median, 25th, and 75th percentiles. The two-tailed Mann–Whitney *U*-test was performed and a *p*-value < 0.05 was considered significant. Nominal *p*-values are given for significant differences (**b**, **d**). ROC analyses for discrimination of study groups for PHA-induced IFN-γ concentrations (**b**) or calculated ratios of PHA/PPD_Mtb_-specific IFN-γ expression (**d**) are shown. Graphs indicate sensitivity and specificity of classification as ROC curves. AUC as well as nominal *p*-values are given. *ROC* Receiver Operator Characteristic, *AUC* Area Under Curve, *ns* not significant

IFN-γ concentrations were measured using Human IFN-γ DuoSet ELISA kit (R&D Systems, USA), following the manufacturer’s instructions. All samples were run in duplicates and analyzed using an Infinite M200 ELISA reader (Tecan, Switzerland). The concentrations were determined from the standard curves using the 4-parametric logistic regression. The IFN-γ concentrations of the non-stimulated samples were subtracted from the corresponding *Mtb* antigen-specific and PHA-induced IFN-γ values for the analyses. We confirmed for results shown in Fig. 1a that the usage of subtracted values did not change the outcome significantly (data not shown). Values below the standard curve were set to 1 pg/ml for depiction and calculations.

### Cytometric bead assay-based quantification of alternative cytokines

The LEGENDplex™ Multi-Analyte Flow Assay kit (Custom Human Assay, Biolegend, USA) was used for the simultaneous detection of different cytokines (i.e., IL-6, IP-10, IL-22, IL-10, GM-CSF, IFN-γ, IL-8) in culture supernatants and plasma samples according to manufacturer’s instructions and as described [12]. Samples were measured using a CytoFlex S flow cytometer (Beckman Coulter, USA). Data were analyzed using the cloud version of the Biolegend LEGENDplex Data Analysis Software (Qognit. Inc). Values below the standard curve were set to 1 pg/ml for depiction and calculations.

### Statistics

All statistical analyses were performed using GraphPad Prism v9 software (GraphPad Software, La Jolla CA, USA). Distribution tests (i.e., Kolmogorov–Smirnov, Shapiro–Wilk) were performed and did not suggest Gaussian distributions for cytokine concentrations. In accordance, we used non-parametric tests throughout. For independent data sets, like the study group comparisons, we performed the Mann–Whitney *U*-test. The Wilcoxon signed-rank test was used for analyses of dependent variables from tuberculosis patients during the time courses. Spearman rank correlation was used to assess co-expression of T-cell and plasma cytokines in both study groups. To evaluate the capacity of differentially expressed cytokines to discriminate tuberculosis patients and contacts we performed Receiver Operating Characteristic (ROC) analysis. We considered a p-value below 0.05 as statistically significant. Graphs were generated using GraphPad Prism version 9.

## Results

### Lower PHA-induced IFN-γ expression in tuberculosis patients with high *M. tuberculosis* sputum burden

We recruited tuberculosis patients (*n* = 55) and healthy contacts (contacts; *n* = 32) from Ghana and compared IFN-γ expression in response to stimulation with *M. tuberculosis* antigens (i.e., ESAT6-CFP10, PPD_Mtb_) and the mitogen PHA. Sputum samples from tuberculosis patients were tested for *M. tuberculosis* burden and cases classified as either sputum positive (TB-SP; *n* = 44) or sputum negative (termed ‘paucibacillary’, TB-PB; *n* = 11) accordingly (for study design details see Supplementary Fig. 1). *Mycobacterium tuberculosis* ESAT6-CFP10 stimulation detected no significant differences between the three study groups (i.e., TB-SP, TB-PB, contacts) and PPD_Mtb_ stimulation induced higher IFN-γ expression in TB-SP compared to contacts (*p* = 0.003) (Fig. 1a). Notably, PHA-induced IFN-γ expression was markedly lower in TB-SP patients as compared to both contacts (*p* < 0.0001) and TB-PB patients (*p* = 0.0096) (Fig. 1a). These results confirmed previous studies and, moreover, suggested association of impaired PHA response with mycobacterial burden. Next, we determined if PHA response differences were capable of discriminating individuals from the study groups. ROC curve analysis detected significant discrimination capacity of PHA responses between TB-SP and both, TB-PB (AUC: 0.75; *p* = 0.011) and contacts (AUC: 0.79; *p* < 0.0001) whereas no significant discrimination was found for TB-PB and contacts AUC: 0.61; *p* = 0.266) (Fig. 1b).

### Moderate association between PHA- and antigen-induced IFN-γ expression in TB-SP

Next, we determined if impaired PHA response at BL was associated with *M. tuberculosis* antigen-specific IFN-γ expression. In this regard, we correlated individual antigen-specific with PHA-induced IFN-γ expression but detected no significant correlations (Supplementary Fig. 2a). Since ratios of *M. tuberculosis* antigen and PHA-induced IFN-γ expression have been described to improve classification of tuberculosis patients [6, 7], we calculated relative values for the study groups. The results of calculated ratios largely resembled differences detectable for PHA (Fig. 1c; Supplementary Fig. 2b). However, we found moderately higher capacity of PPD_Mtb_/PHA ratios to classify TB-SP patients (vs. contacts, AUC: 0.82; Fig. 1d) as compared to PHA-induced IFN-γ alone (Fig. 1b). Moreover, moderate discrimination efficacy was detected between TB-PB and contacts (AUC: 0.74; *p* = 0.019) whereas discrimination between TB-SP and TB-PB was not significant (AUC: 0.67; *p* = 0.081, Fig. 1d). Similar for ESAT6-CFP10/PHA ratios, here only TB-SP and contacts were significantly discriminated (AUC: 0.76; p < 0.0001) but no significance was seen between the other study groups (Supplementary Fig. 2b). We concluded that mainly impaired PHA response classified tuberculosis patients with high pathogen burden.

### Impaired PHA-induced IFN-γ expression in TB-SP normalized early during treatment

**Fig. 2:**
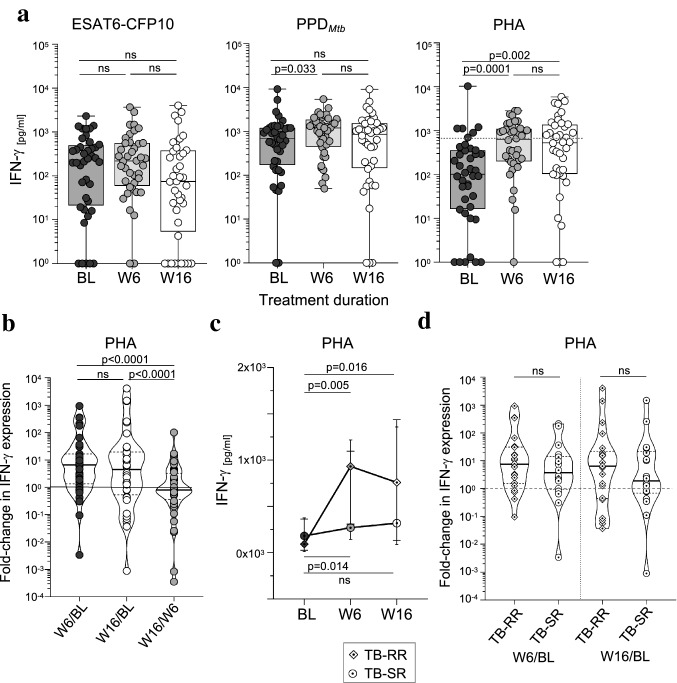
PHA-induced IFN-γ expression normalized early during antimycobacterial treatment. Time course analyses of IFN-γ concentrations after overnight antigen-specific (i.e., PPD_Mtb_, ESAT6-CFP10) or PHA (i.e., phytohemagglutinin) stimulation from tuberculosis patients prior to treatment (baseline, BL; *n* = 40), and 6 weeks (W6; *n* = 40) as well as 16 weeks (W16; *n* = 40) after treatment start. (**a**) Combined symbol/box and whisker graphs depict individual concentrations of IFN-γ and study group medians and ranges. (**b**) Violin plots depict individual fold-changes of PHA-induced IFN-γ expression between W6/BL, W16/BL, and W16/W6 as well as study group distributions including median, 25th, and 75th percentiles. (**c**, **d**) Rapid (TB-RR, *n* = 22) and slow (TB-SR, *n* = 18) treatment responders were compared. (**c**) Connected symbol plots depict the median, 25th, and 75th percentiles of TB-RR and TB-SR at the different time points. The Wilcoxon signed rank test was performed and nominal *p*-values are given in case of significance (*p*-value < 0.05). (**d**) Violin plots depict individual ratios of PHA-induced IFN-γ expression between W6/BL and W16/BL for TB-RR and TB-SR as well as study group distributions including median, 25th, and 75th percentiles. The two-tailed Mann–Whitney *U*-test was performed and nominal p-values are given for significant differences (*p*-value < 0.05)

To investigate the influence of anti-mycobacterial treatment on impaired PHA response, we analyzed antigen- and PHA-induced IFN-γ expression 6 weeks (W6) and 16 weeks (W16) after treatment start (BL) in TB-SP. Antigen-specific T-cell responses hardly changed and only a transient increase of PPD_Mtb_-specific IFN-γ expression was detected at W6 (*p* = 0.033) (Fig. 2a). In contrast, PHA-induced responses in TB-SP rapidly increased during treatment and significantly higher IFN-γ expression was detected at W6 (*p* = 0.0001) and W16 (*p* = 0.002) as compared to BL (Fig. 2a). Median IFN-γ concentrations induced by PHA almost reached contact median values (dotted line) at W6 (Fig. 2a). To compare individual rates of increase and to adjust for interindividual differences in the initial T-cell response, we calculated ratios of PHA-induced IFN-γ expression between the time points. W6/BL and W16/BL ratios were significantly higher than W16/W6 ratios but no difference was seen between W6/BL and W16/BL ratios (Fig. 2b). These results suggested that normalization of T-cell responses to PHA mainly occurred until W6 of antimycobacterial treatment.

### Rapid treatment responders within TB-SP showed marked up-regulation of PHA-induced IFN-γ expression early during treatment

Treatment efficacy was measured by repeated sputum tests at different time points (see methods and Supplementary Fig. 1). On this basis, we classified TB-SP as either ‘rapid’ treatment responders (TB-RR; *n* = 22) or ‘slow’ treatment responders (TB-SR; *n* = 18). BL comparisons detected no significant differences between the subgroups (Supplementary Fig. 3a) and both subgroups showed no differences in antigen-specific responses during treatment (Supplementary Fig. 3b). PHA-induced IFN-γ concentrations increased in both subgroups at W6 (TB-RR, *p* = 0.005; TB-SR, *p* = 0.014) and this effect was more pronounced in TB-RR, who showed significant differences also between BL and W16 (Fig. 2c). Median values between TB-RR and TB-SR differed markedly at W6 (932.5 pg/ml vs. 269.9 pg/ml, respectively; Fig. 2c). Therefore, we compared individual fold-changes between BL and W6 or W16 between the subgroups. Although rapid responders showed higher median ratios for both BL/W6 and BL/W16, no significant differences were detected between the subgroups (Fig. 2d).

### Impaired IFN-γ production by T cells is accompanied by low IL-22 and IL-10 expression induced by PHA

**Fig. 3:**
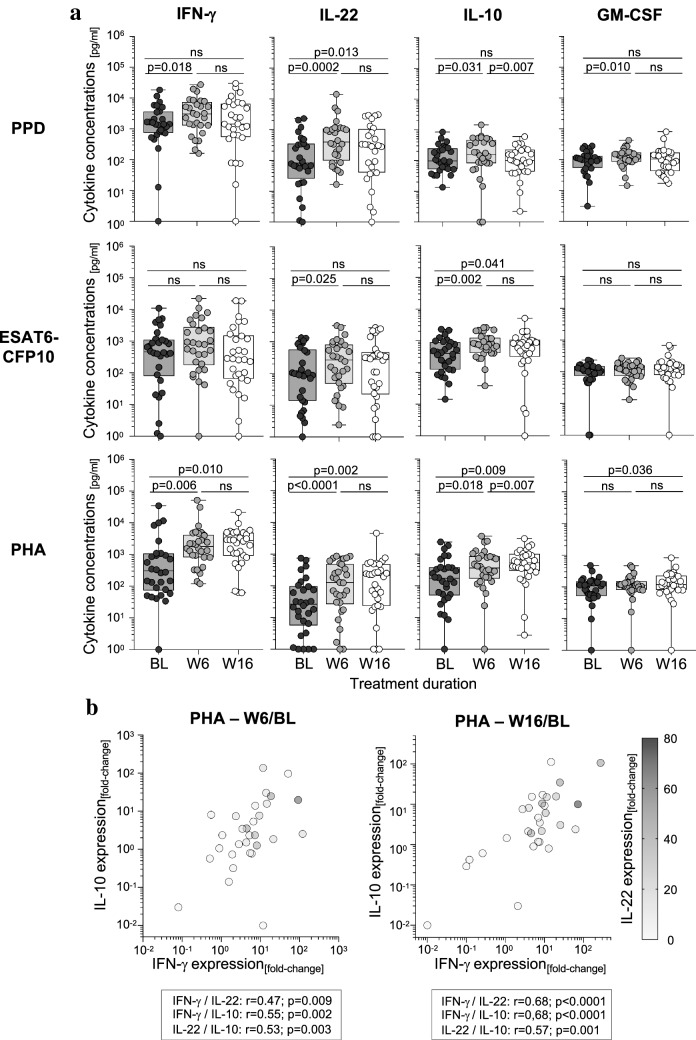
PHA-induced IL-22 and IL-10 normalized early during antimycobacterial treatment and correlated with IFN-γ expression changes. Time course analyses of IFN-γ, IL-22, IL-10, and GM-CSF concentrations after overnight antigen-specific (i.e., PPD_Mtb_, ESAT6-CFP10) or PHA (i.e., phytohemagglutinin) stimulation from patients with tuberculosis prior to treatment (baseline, BL), and 6 weeks (W6) as well as 16 weeks (W16) after treatment start (*n* = 30). (**a**) Combined symbol/box and whisker graphs depict individual concentrations of IFN-γ, IL-22, IL-10 and GM-CSF and study group median values. The Wilcoxon signed rank test was performed and nominal *p*-values are given in case of statistical significance (*p*-value < 0.05). (**b**) Correlations between calculated fold-changes of IFN-γ, IL-22, IL-10 (i.e., W6/BL and W16/BL) are depicted. The Spearman Rank test was applied to determine significance and correlation coefficients (*r*) as well as nominal *p*-values are given

Impaired PHA-induced IFN-γ expression in acute tuberculosis affects the results of immune-based tests for *M. tuberculosis* infection since PHA-stimulated samples are used as positive controls in IGRAs. Hence, we next determined six additional cytokines (i.e., IL-22, IL-10, GM-CSF, IL-6, IL-8, and IP-10) together with IFN-γ in stimulated whole blood supernatants using a cytometric bead assay. All cytokines were detectable after antigen-specific stimulation and increased PPD_Mtb_-specific T-cell responses were detected for IL-22, IL-10, and GM-CSF between BL and W6 (Fig. 3a). ESAT6-CFP10 induced increased response at W6 for IL-22 and IL-10 (as well as for IL-10 at W16) (Fig. 3a). The other cytokines (i.e., IL-6, IL8, IP-10) showed no changes after antigen-specific stimulation under treatment (Supplementary Fig. 4a). Notably, PHA stimulation induced expression pattern of two cytokines, IL-10 and IL-22, with high similarity to IFN-γ (Fig. 3a). Both were significantly increased after PHA stimulation at W6 (IL-22: p < 0.0001; IL-10: *p* = 0.018) and at W16 (IL-22: *p* = 0.002; IL-10: *p* = 0.009) as compared to BL (Fig. 3a). GM-CSF showed no differences between BL and W6 but a moderate increase between BL and W16 (*p* = 0.036) (Fig. 3a). None of the other cytokines (i.e., IP-10, IL-6, IL-8) showed differences in PHA response during treatment (Fig. 3a; Supplementary Fig. 4a). To compare individual cytokine expression changes between the time points, we calculated fold-changes between W6/BL and W16/BL for all candidates. IFN-γ, IL-10 and IL-22 fold-changes showed moderate correlations between BL and W6 (Fig. 3b, left graph) and were strongly correlated between BL and W16 (Fig. 3b, right graph). This indicated concomitant normalization of impaired PHA-induced T-cell expression of IFN-γ, IL-10 and IL-22 after treatment.

### High IL-6 plasma levels are negatively correlated with impaired PHA response of T cells

**Fig. 4:**
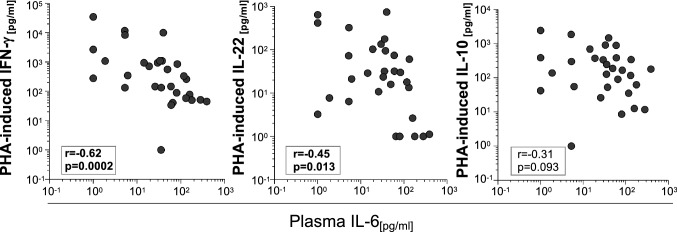
Plasma IL-6 levels correlated with PHA-induced IFN-γ and IL-22 expression at BL. Symbol correlation plots depict plasma IL-6 expression (*x*-axis) and indicated PHA-induced cytokines (*y*-axis) of tuberculosis patients (*n* = 30) at BL. The Spearman Rank test was applied to determine significance and correlation coefficients (*r*) as well as nominal *p*-values are given

Finally, we analyzed cytokine milieu changes in acute tuberculosis as a potential cause for impaired PHA response of T cells. Recently, we described aberrant high plasma levels of specific cytokines (i.e., IL-6, IP-10, IL-10, IL-22) in tuberculosis patients [12]. Since the subgroup of TB-SP was included in both studies (Table 1), we next correlated plasma cytokine concentration with PHA-induced IFN-γ, IL-10 and IL-22 for a subgroup of TB-SP. Whereas plasma concentrations of IP-10, IL-10, and IL-22 were not correlated with PHA responses (Table 2), we detected a marked negative correlation between plasma IL-6 concentrations and PHA-induced IFN-γ (*r* = − 0.62, *p* = 0.0002) (Fig. 4; Table 2). In addition, IL-6 plasma levels were moderately negative correlated with PHA-induced IL-22 (*r* = − 0.45, *p* = 0.013) and a tendency was seen for IL-10 (*r* = − 0.31, *p* = 0.093) (Fig. 4). No significant correlations were seen for plasma IL-6 levels and PPD_Mtb_-specific T-cell responses (Table 3). These results suggested a relation between aberrant high plasma IL-6 levels and impaired PHA response in patients with acute tuberculosis.

**Table 2:** Correlation of plasma cytokines with PHA-induced T-cell cytokines at BL

			PHA-induced at BL		
			IL-10	IFN-γ	IL-22
Plasma cytokines	IL-6	*r*	− 0.3123	− 0.623	− 0.4485
		*p*	0.093	0.0002	0.0129
	IP-10	*r*	0.1462	− 0.0674	0.0543
		*p*	0.4409	0.7234	0.7755
	IL-10	*r*	− 0.0150	− 0.1577	0.0714
		*p*	0.9374	0.4053	0.7076
	IL-22	*r*	0.1136	− 0.2756	− 0.0047
		*p*	0.5502	0.1404	0.9805

*PHA* phytohemagglutinin, *r* correlation coefficient, *p* *p*-value (Spearman rank test), *BL* baseline

**Table 3:** Plasma IL-6 correlation with PPD_Mtb_-induced cytokine expression at BL

	IL-6 vs. IFN-γ	IL-6 vs. IL-10	IL-6 vs. IL-22
Spearman *r*	0.02354	− 0.3641	− 0.05994
*p*-value	0.9054	0.0568	0.7619

*PPD*_*Mtb*_ purified protein derivative of *Mycobacterium tuberculosis*, *BL* baseline, *r* correlation coefficient

## Discussion

The present study identified differences in PHA-induced IFN-γ expression between tuberculosis patients and contacts. Effects of *M. tuberculosis* sputum burden as well as treatment were detected. Novel cytokines (i.e., IL-22, IL-10) reported impaired PHA responses similar to IFN-γ, whereas other cytokines remained unaffected. Furthermore, we identified a negative correlation between high plasma IL-6 concentrations and impaired PHA-induced IFN-γ production of T cells.

The association between aberrant high plasma IL-6 levels and impaired T-cell functions in tuberculosis patients confirmed our previous findings [12–14]. In study groups of tuberculosis patients from the same region in Ghana, we detected constitutive STAT3 phosphorylation in T cells from tuberculosis patients associated with high IL-6 plasma concentrations [14]. The impaired PHA response of T cells seen in the present study may also be caused by constitutive IL-6 signaling although the underlying mechanisms remain elusive. It is important to consider that whole blood stimulation performed in the present study would also detect direct plasma effects on immune cells. Notably, a previous study detected impaired PHA response of tuberculosis patients solely in whole blood and not in PBMC in vitro stimulation [5]. This suggested direct effects of high IL-6 plasma concentrations on T-cell responses of tuberculosis patients.

We detected significant capacity of impaired PHA-induced IFN-γ expression to discriminate between tuberculosis patients and healthy potentially *M. tuberculosis* infected contacts. The general findings were similar with those of some previous studies [7, 9], however, the combination of antigen-specific and PHA-induced effects hardly increased the capacity of classification. This finding was seemingly controversial to other previous studies [6–8] and was likely due to differences in antigen-specific response between the study groups. In the present study, *M. tuberculosis*-specific T-cell responses showed only marginal differences between tuberculosis patients and contacts and this confirmed previous studies from the same region [3, 5]. In contrast, higher antigen-specific IFN-γ expression in contacts as compared to tuberculosis cases were seen in those studies, which detected improved efficacy of ratios for discrimination [7, 8].

We detected impaired PHA response solely in the group *M. tuberculosis* sputum positive cases but not in paucibacillary tuberculosis patients. The conclusion about a potential relationship between *M. tuberculosis* burden and impaired PHA-response was strengthened by the results that treatment normalized PHA-response within 6 weeks, a time frame that led to negative sputum smear/culture tests for approximately half of the patients. The finding of normalization under treatment confirmed recent findings although the time frame was longer [11]. In addition, although we did not detect significant differences between TB-SR and TB-RR under treatment, this did not exclude a relevance of treatment efficacy since the moderate size of subgroups limited the statistical power of this comparison.

Alternative cytokine analyses revealed additional cytokines (i.e., IL-22, IL-10), which showed expression pattern comparable to IFN-γ whereas others did not show any differences during treatment. Against this background, one may conclude that alternative cytokines, like the well-investigated candidate IP-10 can be used to circumvent impaired IFN-γ response. IP-10 showed IFN-γ-similar or even improved capacity to detect *M. tuberculosis* infection in previous studies [15–18]. Other candidates, like IL-6, have been shown to have an even higher sensitivity to detect T-cell responses in tuberculosis patients including paucibacillary cases [3, 4, 19–21]. The cellular origin of cytokines cannot be deduced in this assay and, hence, alternative sources due to indirect activation of other immune cells by T cells is possible.

In summary, this study contributed to the knowledge about T-cell pathology in tuberculosis and adds to the identification of biomarkers for treatment monitoring and diagnosis in tuberculosis.

## Supplementary Information

Below is the link to the electronic supplementary material.

Supplementary file1 (PDF 1492 KB)

## Data Availability

Any dataset is available from the authors upon reasonable request.
