# Supplementary material for: Impaired T-cell response to phytohemagglutinin (PHA) in tuberculosis patients is associated with high IL-6 plasma levels and normalizes early during anti-mycobacterial treatment
Source: Infection. 2023 Jan 18;51(4):1013–23. doi: 10.1007/s15010-023-01977-1 (PMC10352402; doi:10.1007/s15010-023-01977-1)
Supplement: Supplementary file 1 — Supplementary file1 (PDF 1492 KB) [file 15010_2023_1977_MOESM1_ESM.pdf]

Supplementary Figure 1

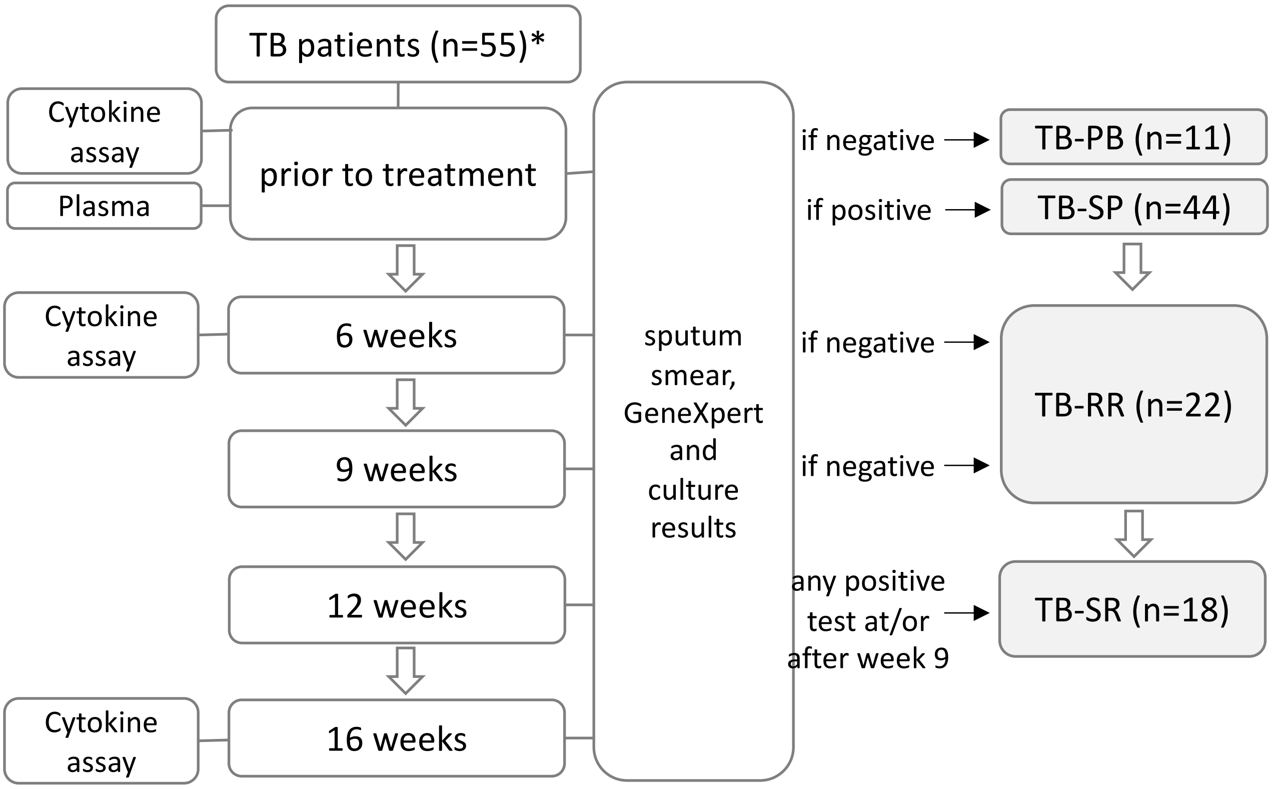

Recruitment scheme for *in vitro* stimulation (cytokine assay), plasma, sputum analyses, and criteria for classification of tuberculosis patient subgroups. TB-SP, sputum positive tuberculosis patients; TB-PB, paucibacillary Tuberculosis patients; TB-RR, rapid treatment responder; TB-SR, slow treatment responder.

Supplementary Figure 2

a

b

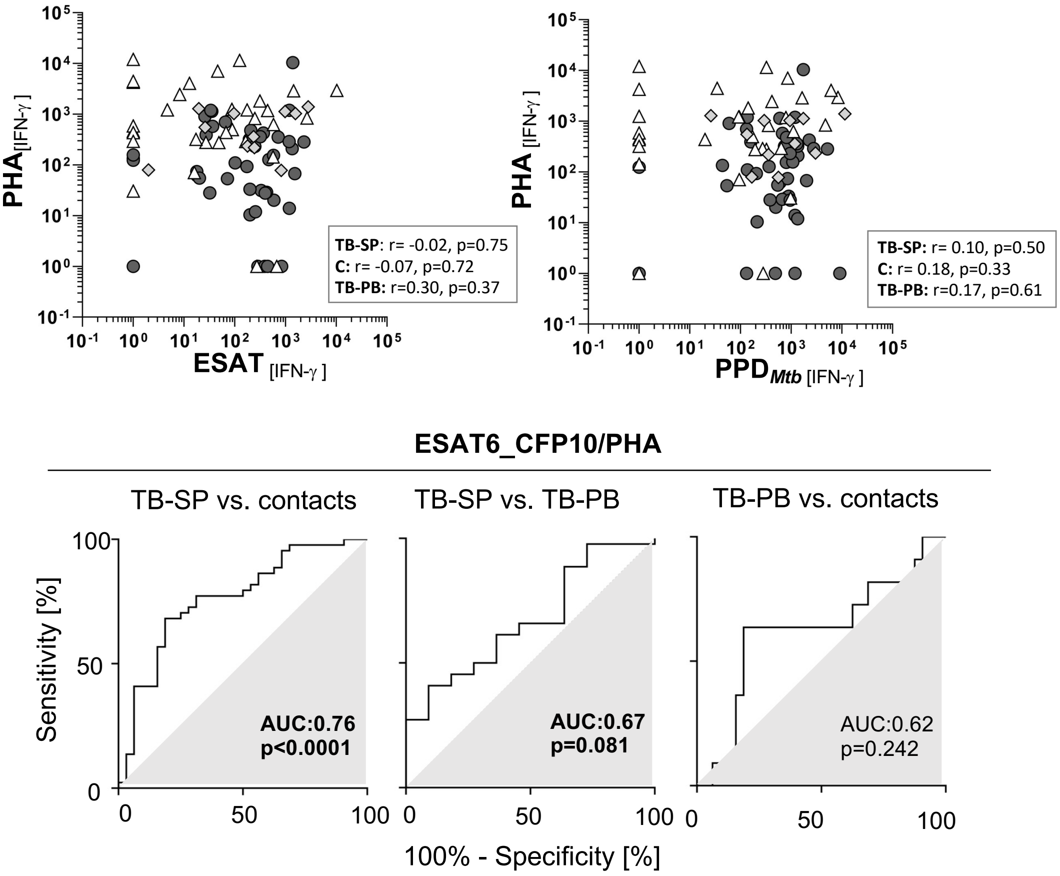

Antigen-specific (i.e., PPD_{Mtb}, ESAT6-CFP10) and PHA (i.e., phytohemagglutinin) induced IFN-γ concentrations of tuberculosis patients with high (TB-SP; dark grey circles; n=44) and low (TB-PB; bright grey diamonds; n=11) *M. tuberculosis* burden as well as contacts (open triangles; n=32) are depicted. (**a**) Correlation plots of PHA vs. ESAT6-CFP10 (left graph) and vs. PPD_{Mtb} (right graph) are shown. The Spearman Rank test was applied to determine significance and correlation coefficients (r) as well as nominal p-values are given. (**b**) ROC analyses for discrimination of study groups for calculated ratios of PHA and ESAT6-CFP10-specific IFN-γ expression are shown. Graphs indicate sensitivity and specificity of classification as ROC curves. AUC as well as nominal p-values are given. ROC: Receiver Operator Characteristic; AUC: Area Under Curve. ns: not significant.

Supplementary Figure 3

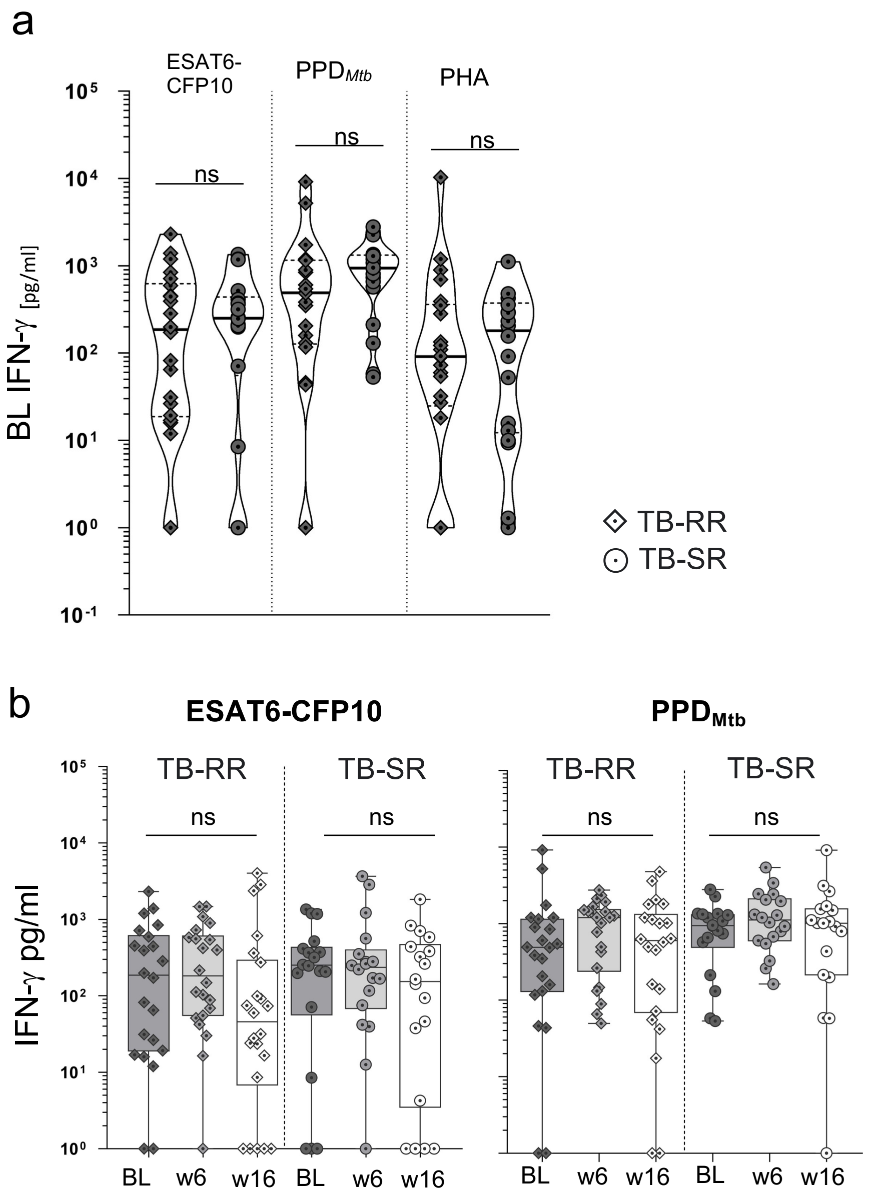

Antigen-specific (i.e., PPD_{Mtb}, ESAT6-CFP10) and PHA-induced IFN-γ concentrations of tuberculosis patients with rapid (TB-RR, n=22) and slow (TB-SR, n=18) treatment response were compared

at different time points after treatment start. (**a**) Violin plots depict individual concentrations of IFN-γ as well as study group distributions including median, 25, and 75 percentiles. The two-tailed Mann-Whitney U-test was performed and a p-value <0.05 was considered significant. (**b**) Combined symbol/box and whisker graphs depict individual concentrations of IFN-γ and study group median values. The Wilcoxon signed rank test was performed for time courses comparisons. A p-value <0.05 was considered significant. ns: not significant.

Supplementary Figure 4

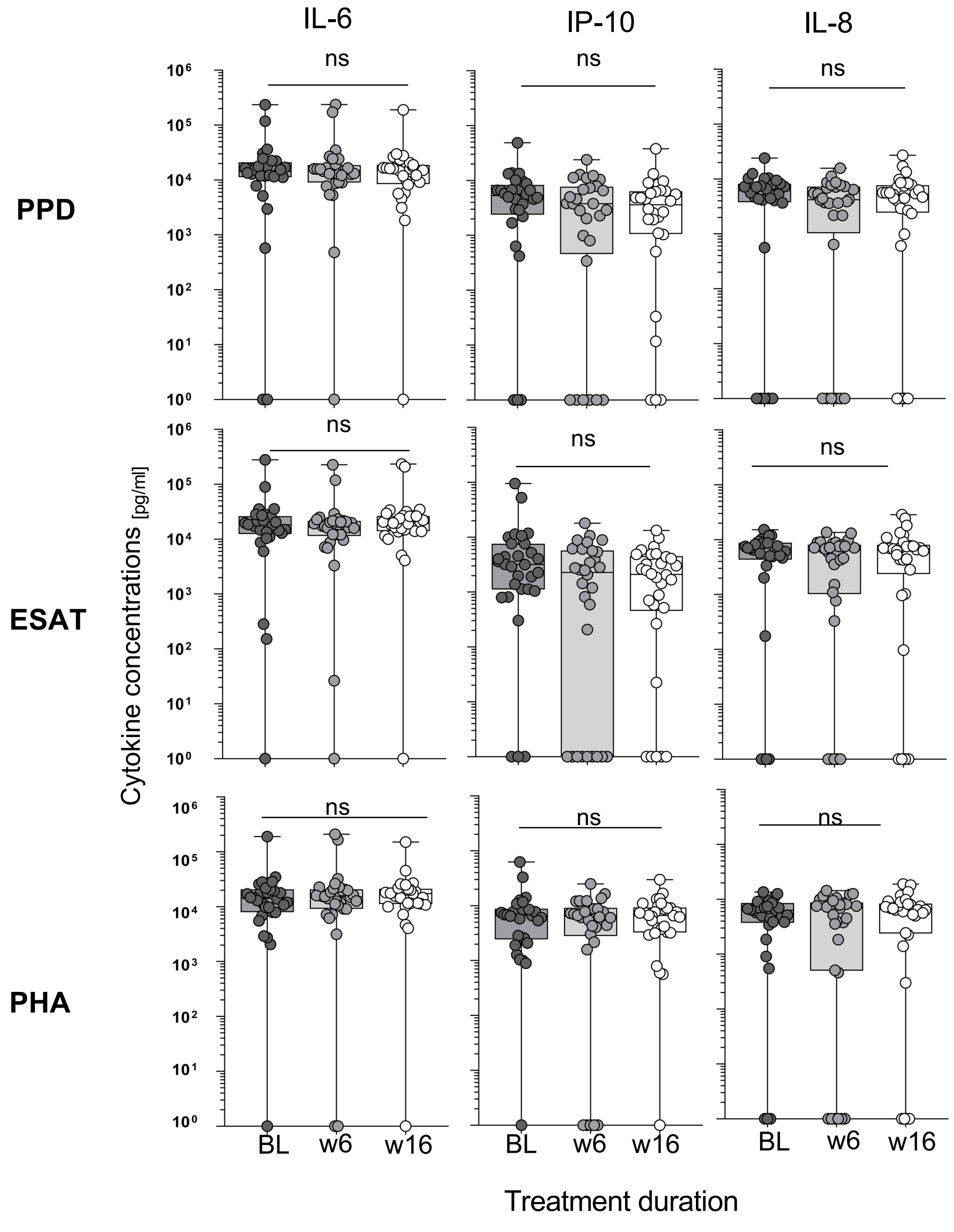

Time course analyses of IL-6, IP-10, and IL-8 concentrations after overnight antigen-specific (i.e., PPD_{Mtb}, ESAT6-CFP10) or PHA (i.e., phytohemagglutinin) stimulation from patients with tuberculosis prior to treatment (baseline, BL), and six weeks (W6) as well as 16 weeks (W16) after treatment start. Combined symbol/box and whisker graphs depict individual concentrations of IL-6, IP-10, and IL-8 and study group median values. The Wilcoxon signed rank test was performed and nominal p-values are given in case of statistical significance (p-value <0.05).

Supplementary Table 1: Tuberculosis patients diagnostic tests at baseline and classification

TB-SP				
**Patient ID**	**Sputum smear**	**Sputum culture**	**GeneXpert**	**Treatment response**
1	pos	pos	pos	TB-SR
2	pos	pos	pos	TB-RR
3	pos	pos	pos	TB-RR
4	pos	pos	pos	TB-SR
5	pos	neg	pos	TB-RR
6	pos	neg	nd	TB-RR
7	pos	pos	pos	TB-SR
8	pos	neg	nd	TB-RR
9	pos	pos	pos	TB-RR
10	nd	pos	nd	TB-SR
11	pos	pos	pos	nd*
12	nd	pos	pos	TB-RR
13	nd	pos	pos	TB-RR
14	pos	pos	pos	TB-SR
15	pos	pos	pos	TB-SR
16	pos	pos	pos	TB-RR
17	nd	pos	pos	TB-SR
18	pos	neg	pos	TB-SR
19	pos	neg	pos	TB-RR
20	pos	pos	pos	TB-RR
21	nd	pos	pos	TB-RR
22	nd	pos	pos	nd*
23	pos	pos	pos	nd*
24	pos	pos	pos	TB-RR
25	pos	pos	pos	TB-SR
26	pos	pos	pos	TB-SR
27	pos	pos	pos	TB-RR
28	nd	pos	pos	TB-RR
29	pos	pos	pos	TB-SR
30	pos	pos	pos	TB-SR
31	pos	pos	pos	TB-RR
32	nd	pos	pos	TB-SR
33	nd	pos	pos	TB-SR
34	nd	pos	pos	TB-RR
35	pos	pos	pos	TB-RR
36	pos	pos	pos	TB-SR
37	pos	pos	nd	TB-RR
38	pos	pos	pos	nd*
39	pos	pos	pos	TB-RR
40	nd	pos	nd	TB-SR
41	pos	pos	pos	TB-RR
42	pos	pos	pos	TB-SR
43	pos	pos	pos	TB-SR
44	pos	pos	pos	TB-RR

TB-PB			
**Patient ID**	**Sputum smear**	**Sputum culture**	**GeneXpert**
1	neg	neg	pos
2	neg	neg	nd**
3	neg	neg	pos
4	neg	neg	pos
5	neg	neg	nd**
6	neg	neg	neg**
7	neg	neg	pos
8	neg	neg	nd**
9	neg	neg	pos
10	neg	neg	pos
11	neg	neg	pos

TB-SP: Sputum positive tuberculosis patients at baseline
TB-PB: Sputum negative tuberculosis patients at baseline
ID: identifier
pos: positive
neg: negative
nd: not done
* excluded from analyses, not all time points available
** positive sputum results during treatment, i.e., ID2: positive smear/sputum culture at w16; ID5: positive sputum smear at w6; ID6: positive sputum culture at w12; ID8: positive sputum culture at w6
